# Profiling mycobacterial communities in pulmonary nontuberculous mycobacterial disease

**DOI:** 10.1371/journal.pone.0208018

**Published:** 2018-12-11

**Authors:** Steven A. Cowman, Phillip James, Robert Wilson, William O. C. Cookson, Miriam F. Moffatt, Michael R. Loebinger

**Affiliations:** 1 National Heart and Lung Institute, Imperial College London, London, United Kingdom; 2 Host Defence Unit, Royal Brompton Hospital, London, United Kingdom; National Institute of Infectious Diseases, JAPAN

## Abstract

The diagnosis of pulmonary non-tuberculous mycobacterial disease (pNTM) is dependent on the isolation of NTM in culture, which is prone to overgrowth and contamination and may not capture the diversity of mycobacteria present, including rare or unidentified species. This study aimed to develop a culture independent method of detecting and identifying mycobacteria from sputum samples using partial sequencing of the hsp65 gene. DNA was extracted from sputum samples from subjects with pNTM and disease controls. Multiplexed partial sequencing of the hsp65 gene was performed using the Illumina MiSeq and custom primers. A reference database of hsp65 sequences was created for taxonomy assignment. Sequencing results were obtained from 42 subjects (31 cases, 11 controls). Mycobacterial sequences were identified in all subjects. In 90.5% of samples more than one species was found (median 5.5). The species isolated in culture was detected by sequencing in 81% of subjects and was the most abundant species in 62%. The sequencing of NTM from clinical samples reveals a far greater diversity than conventional culture and suggests NTM are present as communities rather than a single species. NTM were found to be present even in the absence of isolation in culture or clinical disease.

## Introduction

Pulmonary infection with non-tuberculous mycobacteria (NTM) is a challenging disease which is becoming increasingly prevalent. It is associated with a high mortality, and treatment is prolonged, expensive and frequently poorly tolerated [1,2]. The diagnosis of NTM infection relies upon the isolation of NTM in culture which is challenging due to the overgrowth of other co-pathogens, such as *Pseudomonas*, contamination from environmental mycobacteria and failure to grow due to fastidious requirements, as well as the time and labour involved.

Molecular techniques utilising nucleotide amplification have been used directly on clinical samples in TB infection and have become established in clinical practice [3]. Their use in NTM disease, however, is currently confined to the identification of species previously cultured from clinical samples. As well as overcoming the limitations of culture based techniques, the sequencing of mycobacteria directly from a sample has the potential to capture the diversity of mycobacterial species present including rare and as yet unidentified species. This may be valuable, as the isolation of multiple species is not uncommon and may pose a therapeutic challenge [4,5]. Molecular techniques have been successfully employed in both environmental [6–8] and more recently in human samples [9].

The aim of this study was to employ next generation sequencing of the *hsp65* gene to characterise mycobacterial communities in the lung directly from sputum samples.

## Materials and methods

Written consent was gained from all participants and the study was approved by the London - Chelsea Research Ethics Committee (reference 12/LO/1034). Subjects were recruited from the outpatient department of the Royal Brompton Hospital between September 2012 and November 2013. Cases were enrolled if they met ATS/IDSA 2007 criteria [10] and had non-cystic fibrosis bronchiectasis, chronic obstructive pulmonary disease (COPD) or no underlying lung disease prior to their diagnosis of NTM disease. Controls had non-cystic fibrosis bronchiectasis or COPD with no clinical or radiological evidence of NTM infection, previous negative sputum mycobacterial cultures and a negative sputum mycobacterial culture on enrolment. Exclusion criteria were: pregnancy, immunosuppression (other than oral corticosteroids), and current malignancy or tuberculosis.

Participants underwent clinical assessment and pulmonary function testing. If not performed within the previous six months high resolution computed tomography (HRCT) was performed. Spontaneously expectorated sputum was stored at -80 degrees Celsius for DNA extraction as well as undergoing phenol auramine microscopy and culture for bacteria, mycobacteria and fungi.

Barcoded primers were designed for use with the MiSeq benchtop sequencer (Illumina, San Diego, CA, USA) as previously described [11] using the TB11/TB12 primer pair, which amplify a 401bp region of the mycobacterial *hsp65* gene [12]. This target was chosen as the amplicon length is suitable for the MiSeq platform, and enables clear differentiation between *M*. *abscessus* and *M*. *chelonae* [13] as well as differentiation between *M*. *abscessus* spp. *abscessus* and ssp. *bolletii/massiliense* which are identical by partial sequencing of the 16S rRNA gene [14–16].

As a positive control, a mock community was created consisting of *hsp65* amplicons from 16 NTM typestrains cloned, sequenced and pooled in equal abundance. A reference database was created consisting of all mycobacterial *hsp65* sequences present in the National Center for Biotechnology Information (NCBI) GenBank (http://www.ncbi.nlm.nih.gov/nuccore/) in addition to the sequences of the NTM typestrains of the mock community and non-mycobacterial sequences which also may be amplified by the TB11/TB12 primer pair, identified using NCBI primer-BLAST (https://www.ncbi.nlm.nih.gov/tools/primer-blast/).

For each patient a 300μl aliquot of stored sputum underwent bead-beating and DNA was extracted using hexadecyltrimethylammonium bromide and phenol: chloroform:isoamylalcohol (see supporting information for further information). As a control, molecular-grade water was collected in the same receptacles as used for sputum and subjected to the same storage and DNA extraction protocol. Barcoded *hsp65* sequences were amplified from each DNA sample in quadruplicate and libraries prepared and sequenced using the MiSeq benchtop sequencer and MiSeq v3 600 cycle reagents (Illumina).

Sequence processing was performed using the QIIME pipeline 1.8.0 [17] applying a 99% similarity for Operational taxonomic unit (OTU) picking. The resulting data was imported into the R environment version 3.1.2 [18] where all downstream analysis was performed. The relative abundance of taxa between groups were compared using the Wilcoxon rank sum test using the Benjamini-Hochberg correction for multiple testing. Diversity metrics were calculated using the phyloseq and vegan packages [19,20] and compared between groups using the t-test and correlated with clinical variables using Pearson correlation. Differences in community structure were tested using permuted analysis of variance (PERMANOVA) in the vegan package. A *P* value of < 0.05 was considered significant.

Full details of the *hsp65* database and mock community creation, DNA extraction, library preparation, sequencing and the processing of sequence data can be found in the supporting information. Sequence data are available from the European Nucleotide Archive under accession number PRJEB22153.

## Results

Sputum was collected for DNA extraction from 56 eligible subjects. A total of 4.84 million joined sequences were obtained, leading to 4.16 million sequences available for analysis following processing and quality control (for more detail see the supporting information). Samples with a low depth of sequencing (under 5,000 sequences) were removed (N = 14) leaving 42 subjects for analysis with a median depth of sequencing of 80,960 sequences per sample. The clinical characteristics of the final study population are shown in Table 1. All controls were smear and culture negative and had provided at least one prior negative mycobacterial culture (median 4 samples, range 1 to 15).

**Table 1:** Clinical characteristics of the study population.

	Cases N = 31	Controls N = 11
**Age (years)**	65.77 (±11.32)	65.58 (±4.78)
**Male sex**	12 (38.7%)	6 (54.5%)
**Ex- or current smoker**	15 (48.4%)	6 (54.5%)
**Underlying diagnosis**		
COPD	5 (16.1%)	0
Bronchiectasis	23 (74.2%)	11 (100%)
No underlying lung disease	3 (9.7%)	0
**FEV1% predicted**	52.85 (±29.30)	79.19 (±16.67)
**FVC % predicted**	89.96 (±32.01)	92.73 (±15.29)
**Prophylactic antibiotics**	7 (22.6%)	5 (45.5%)
**Oral steroid**	1 (3.2%)	2 (18.2%)
**NTM treatment**	7 (22.6%)	-
**NTM species**		
*M*. *abscessus*	4 (12.9%)	-
*M*. *avium* complex (MAC)	18 (58.1%)	-
*M*. *fortuitum*	1 (3.2%)	-
*M*. *kansasii*	5 (16.1%)	-
*M*. *malmoense*	1 (3.2%)	-
*M*. *simiae*	1 (3.2%)	-
*M*. *xenopi*	1 (3.2%)	-

Continuous variables are expressed as mean +/- standard deviation, categorical variables as count and percentage. NTM species refers to the species isolated in culture leading to the original diagnosis of pulmonary NTM disease.

All species within the mock community were detected. The median difference between the observed proportional abundance of each species and the expected proportion was 2.7% (range 0.2% to 5.5%) with *M*. *xenopi* and *M*. *tuberculosis* complex being the most overrepresented and *M*. *abscessus subsp*. *abscessus* and *M*. *peregrinum* being the most underrepresented (S2 Fig). There was no evidence of contamination in the negative extraction controls or the negative PCR control.

### Genus level

Mycobacterial sequences were detected in all samples, although only 6.23% of all sequences were from mycobacteria and the median proportion of mycobacterial sequences in each sample was 0.15% (S3 Table and S4 Fig). The most abundant genus was Actinomyces, accounting for 51.5% of all sequences. Samples tended to be dominated by one genus and in 38 samples one genus represented over 50% of all sequences. In two subjects Mycobacterium was the most abundant genus; both were culture positive for *Mycobaterium avium* complex (MAC) and *M*. *abscessus* respectively. There was no significant difference in the median relative abundance of mycobacteria in cases compared with controls (0.19% vs 0.02%, *P* = 0.65). There was no significant correlation between mycobacterial abundance and *hsp65* amplicon concentration (Spearman rho -0.18 *P* = 0.255, see S3 Fig in the supporting information).

### Species level

All sequences from the genus Mycobacterium (N = 258,421) were selected for further analysis. The most common species was *M*. *avium*, followed by *M*. *abscessus* subsp. *bolletii* and *M*. *simiae* (Table 2). In 32 samples the most abundant species constituted over 50% of all mycobacterial sequences. More than one species was identified in 38 (90.5%) of samples. The median number of species present was 5.5 (range 1 to 19). Details of sequencing for each subject are shown in Table 3.

**Table 2:** The abundance of mycobacterial species discovered by sequencing.

Species	Mean proportion reads per sample	No. subjects where present	No. subjects where most abundant	Number of OTUs
*M*. *avium*	35.01%	33	14	107
*M*. *abscessus* subsp. *bolletii*	17.44%	28	8	28
*M*. *simiae*	13.67%	27	8	15
*M*. *abscessus* subsp. *abscessus*	6.55%	23	3	35
*M*. *xenopi*	5.32%	13	2	12
*M*. *intracellulare chimaera*	4.40%	22	3	3
*M*. *intracellulare*	4.16%	25	1	37
*M*. *kansasii*	3.81%	16	2	5
*M*. *arupense*	1.37%	13	0	11
*M*. *chelonae*	1.08%	13	1	9
*M*. *rhodesiae*	0.99%	9	0	8
*M*. *fortuitum*	0.97%	11	0	13
*M*. *gordonae*	0.94%	7	0	4
*M*. *psychrotolerans*	0.89%	13	0	4
*M*. *malmoense*	0.71%	13	0	13
*M*. *peregrinum*	0.58%	9	0	7
*M*. *tuberculosis* complex	0.58%	11	0	20
*M*. *neoaurum*	0.52%	3	0	1
*M*. *szulgai*	0.39%	13	0	4
*M*. *kubicae*	0.02%	2	0	1
*M*. *abscessus* complex	0.01%	1	0	1
Unidentified	0.58%	10	0	17

**Table 3:** Sequencing and culture results for each subject.

ID	Group	Diagnosed species	No. mycobacterial sequences	No. mycobacterial OTUs	Most abundant species by sequencing	Cultured species	Cultured species detected by sequencing
282	Case	*M*. *abscessus*	156074	57	*M*. *abscessus subsp*. *bolletii*	*M*. *abscessus*	Yes
272	Case	MAC	75301	173	*M*. *avium*	MAC	Yes
238	Case	MAC	11622	119	*M*. *avium*	MAC	Yes
212	Case	*M*. *abscessus*	4092	74	*M*. *abscessus subsp*. *abscessus*	*M*. *abscessus*	Yes
216	Case	*M*. *abscessus*	3745	39	*M*. *abscessus subsp*. *abscessus*	MAC	Yes
291	Case	*M*. simiae*	1648	55	*M*. *simiae*	No sample	-
248	Case	*M*. *fortuitum*	1410	21	*M*. *xenopi*	No growth	-
270	Case	MAC	557	23	*M*. *avium*	No growth	-
269	Case	MAC	441	25	*M*. *avium*	MAC	Yes
218	Case	MAC	310	27	*M*. *avium*	MAC	Yes
226	Case	*M*. *kansasii*	247	10	*M*. *avium*	*M*. *kansasii*	Yes
277	Case	*M*. *kansasii*	204	15	*M*. *kansasii*	*M*. *kansasii*	Yes
301	Case	MAC	199	15	*M*. *simiae*	No sample	-
237	Case	*M*. *malmoense*	165	3	*M*. *simiae*	*M*. *xenopi*	No
205	Case	MAC	133	26	*M*. *abscessus subsp*. *bolletii*	No growth	-
268	Case	MAC	119	30	*M*. *intracellulare chimaera*	MAC	Yes
271	Case	*M*. *kansasii*	102	6	*M*. *kansasii*	M. kansasii	Yes
229	Case	MAC	101	29	*M*. *simiae*	MAC	Yes
249	Case	*M*. *kansasii*	97	11	*M*. *simiae*	*M*. *kansasii*	No
266	Case	*M*. *kansasii*	67	5	*M*. *abscessus subsp*. *abscessus*	*M*. *kansasii*	Yes
294	Case	*M*. *abscessus*	57	9	*M*. *avium*	No growth	-
214	Case	MAC	54	4	*M*. *xenopi*	*M*. *xenopi*	Yes
276	Case	*M*. *xenopi*	47	2	*M*. *abscessus subsp*. *bolletii*	*M*. *xenopi*	No
250	Case	MAC	32	3	*M*. *abscessus subsp*. *bolletii*	No growth	-
245	Case	MAC	17	9	*M*. *avium*	MAC	Yes
235	Case	MAC	9	6	*M*. *intracellulare chimaera*	No growth	-
241	Case	MAC	9	5	*M*. *intracellulare chimaera*	MAC	Yes
299	Case	MAC	7	3	*M*. *avium*	No growth	-
224	Case	MAC	6	2	*M*. *abscessus subsp*. *bolletii*	No growth	-
263	Case	MAC	2	1	*M*. *avium*	MAC	Yes
222	Case	MAC	1	1	*M*. *avium*	*M*. *abscessus*	No
303	Control		468	15	*M*. *abscessus subsp*. *bolletii*	No growth	-
220	Control		209	50	*M*. *abscessus subsp*. *bolletii*	No growth	-
306	Control		176	47	*M*. *simiae*	No growth	-
217	Control		167	22	*M*. *avium*	No growth	-
231	Control		143	12	*M*. *simiae*	No growth	-
210	Control		140	15	*M*. *avium*	No growth	-
302	Control		111	38	*M*. *abscessus subsp*. *bolletii*	No growth	-
211	Control		68	9	*M*. *simiae*	No growth	-
305	Control		31	7	*M*. *avium*	No growth	-
225	Control		26	12	*M*. *intracellulare*	No growth	-
304	Control		7	6	*M*. *chelonae*	No growth	-

Diagnosed species refers to the species isolated in culture leading to the original diagnosis of pulmonary NTM disease. Cultured species refers to the NTM species isolated from the study sample at the time of sequencing. ID = sample identifier.

* isolated MAC prior to *M*. *simiae* but not re-isolated for 10 years

**Fig 1:**
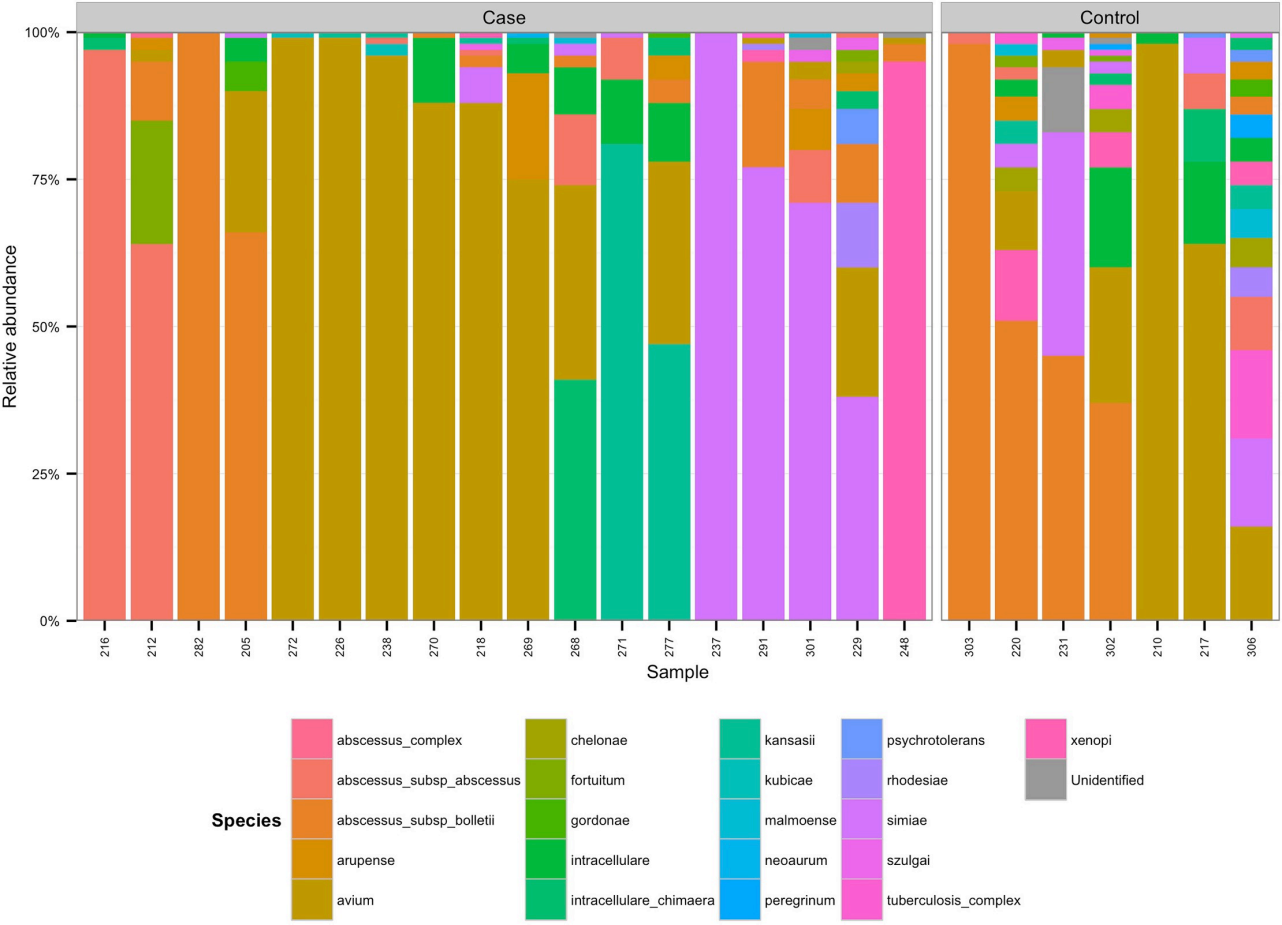
Mycobacterial community structure in samples with more than 100 mycobacterial sequences. X-axis = samples, Y-axis = relative abundance, colours = species, left panel = cases, right panel = controls.

To allow comparison at the community level between subjects with varying depth of sequencing, samples were rarefied to a depth of 100 sequences leaving 25 subjects (18 cases and 7 controls) for analysis. The community structure of each subject is shown in Fig 1. After rarefaction the majority of subjects (92%) had more than one species present (median 5, range 2 to 17). There were no significant differences in the abundance of any NTM species between cases and controls, and no difference in community structure by PERMANOVA.

Exploration of diversity (S5, S6 and S7 Figs) revealed a significant reduction in diversity as measured by Shannon and Pielou’s indices in NTM cases compared to controls. Within NTM cases alone, Shannon index and observed richness were higher in those receiving treatment for NTM, and Shannon and Pielou’s indices were positively correlated with forced expiratory volume in 1 second.

### Comparison with sputum culture

The cultured NTM species was detected by sequencing in 17 (81%) of the 21 culture positive samples (Table 3). NTM were detected by sequencing in all of the 19 culture-negative samples. The median proportion of mycobacterial sequences was lower in the four discordant samples where the isolated species was not detected by sequencing (0.006%) compared to the concordant samples where the isolated species was also detected by sequencing (0.12%) or the culture negative samples (0.31%), although this difference did not reach significance (S8 Fig, Kruskal-Wallis *P* = 0.054).

In 13 of the 21 cases (62%) with culture-positive sputum the isolated species was also the most abundant species found by sequencing. Agreement was highest for *M*. *kansasii*, which was the most abundant species in both culture positive subjects, and lowest for *M*. *simiae*, which was not cultured from any of the five subjects in which it was the most abundant species by sequencing. No sputum sample isolated more than one NTM species by culture. However, in 12 cases (39%) more than one NTM species had been previously isolated at the time of study enrolment (S4 Table).

## Discussion

This study has demonstrated the utility of next generation sequencing with multiplexed barcoded paired-end primers to sequence mycobacterial hsp65 directly from sputum samples. The majority of sequences were identified to the species level. The study is the first to provide a profile of mycobacterial communities within the lung.

The use of a mock community confirms the ability to detect a broad range of NTM species and allows assessment of amplification bias. Contamination is an important concern in microbiome studies [21] and the use of extraction controls mirroring the processing of sputum samples provides reassurance that the findings are unlikely to be the result of contamination.

Mycobacterial sequences were recovered from every sample in both cases and controls, regardless of their growth in culture or the presence of clinical disease. It has long been appreciated that mycobacteria may be cultured from subjects in the absence of disease. This has led to the development of microbiological criteria for the diagnosis of NTM disease [10] which are not met by around half of subjects in whom NTM are isolated [22,23]. This has been attributed to contamination or ‘casual’ isolation of transiently inhaled organisms, however the data from this present study suggest that this isolation may reflect the true recovery of NTM from the respiratory tract, and that sputum culture may in fact lack sensitivity to the presence of NTM. However, the detection of microbial DNA by sequencing does not necessarily indicate the presence of viable organisms and it is also possible that the recovery of some sequences, particularly those from species not known to cause disease, may originate from non-viable inhaled organisms. The control group was composed of subjects with bronchiectasis, a known risk factor for NTM disease, and it may be that the ubiquitous finding of NTM is restricted to this particular clinical phenotype. A useful avenue for further work will be to determine if NTM are present in the lungs of subjects with other chronic lung diseases, or even in healthy individuals.

Despite their ubiquity in the environment, mycobacteria have not been frequently reported in previous studies of the lung microbiome. Their abundance may however be underestimated for a variety of reasons. In contrast to many other taxa most mycobacteria possess only a single copy of the 16S rRNA gene [24], their protective cell wall is particularly resistant to lysis for DNA extraction [25] and a number of primers targeting the 16S rRNA gene do not comprehensively cover the genus [26].

Several culture-based studies have found that the isolation of more than one NTM species from the same individual is not uncommon [4,5,22] and another key finding of this study is that in nearly all samples mycobacteria were present as a community rather than a single species. This was not detected by sputum culture which failed to detect more than one species in any individual, although the historic isolation of more than one species was seen in over one-third of cases. This is important finding especially as the current paradigm of NTM infection is that of infection with a single species, which along with the radiological pattern of disease defines that individual’s diagnosis and governs their treatment.

There was a tendency for one NTM species to dominate the community and this was more marked in NTM cases. Although not statistically significant, cases had lower alpha diversity indices than controls. Diversity indices were also lower in cases with poor lung function but higher in those treated for NTM. This may represent the expansion of one pathogenic species in subjects who develop clinical NTM disease. The differences however were small and dominance by a single species was still frequently seen in controls without disease. In four samples the cultured NTM species was not detected by sequencing; this is highly likely to be due to the very low numbers of mycobacterial sequences (between 1 and 165) recovered from these samples.

The most abundant mycobacteria species sequenced was *M*. *avium*, in keeping with the results of conventional culture in the study cohort and other published culture-based studies [23,27–29]. Surprisingly the second most common species was *M*. *simiae*, despite not being detected by culture in any sample and not being a commonly encountered species in the UK or the rest of Europe. The reason for this finding is unclear. There was no clear bias seen in favour of *M*. *simiae* amplification in the mock community and the bacterium is readily isolated by conventional culture techniques with no special requirements.

Measures of mycobacterial diversity were lower in NTM cases compared to controls, and within NTM cases higher diversity was associated with better lung function and with treatment directed against NTM. These findings parallel those seen in studies of the bacterial microbiome in respiratory disease which have repeatedly found lower bacterial diversity to be associated with more severe disease [30–36].

The only comparable culture independent study employing human clinical samples was reported by Macovei and colleagues [9] who used a nested PCR targeting mycobacteria to perform partial sequencing of the 16S rRNA gene on samples from the oral cavity and upper respiratory tract of healthy subjects. In keeping with the present study multiple taxa of NTM were found to be present in every subject.

The principal limitation of the present study is the recovery of a large amount of non-mycobacterial sequences, which had the effect of significantly reducing the depth of mycobacterial sequencing. Consequently the data may underestimate the true diversity of mycobacteria present, and may fail to detect mycobacteria in some samples with low sequencing depth. The amplification of non-mycobacterial sequences also meant that the primers could not be used for quantitative PCR to estimate the absolute abundance of mycobacteria in the samples. The mock community data also suggests that there is some variation in amplification between species which may influence estimates of community structure.

Sputum samples are subject to oral contamination and may originate from anywhere within the lung, and so potentially may not reflect the community composition at the site of localised disease. However bronchoscopy would not be feasible in many of the study participants who have advanced pulmonary disease and other comorbidities, and bronchoscopic sampling is also not without risk of contamination [37]. Given the known presence of NTM in the oral cavity [9] contamination is a potential source of NTM sequences in the sputum, however in comparison to sputum oral secretions are likely to have contributed only a small fraction of the total biomass sequenced, and reassuringly there was no relationship between amplicon concentration and mycobacterial abundance, which has been shown to be a marker of contamination in 16S data [38].

In the present study subjects were sampled at a single time point and only one sputum sample was used for sequencing. Future work will analyse successive sputum samples at a single time point to assess the reproducibility of the community structure, as well as longitudinal sampling to characterise the resilience and responsiveness of the community to changes in clinical state over time.

Finally, the study group was heterogeneous and composed of individuals with different pulmonary conditions receiving different treatments, including antibiotics and therapy for NTM. There are differences between NTM cases and the control group, notably the use of NTM therapy which was by definition absent in the control group, and a lack of subjects with COPD in the control group as sufficient sputum could not be obtained for sequencing in these subjects. These differences may confound analyses between groups. Furthermore, the small sample size means the analysis of diversity metrics is underpowered. Therefore these particular findings should be treated as exploratory.

## Conclusions

This study represents the first use of multiplexed next generation partial sequencing of the *hsp65* gene to characterise mycobacterial communities in the lung. This found that NTM were present in all study subjects regardless of their isolation by conventional culture or the presence of clinical NTM disease, and that multiple species of NTM were usually present in the same individual.

## Supporting information

S1 File Supporting information. (DOCX)

S1 Table NTM typestrains included in the mock community. (DOCX)

S2 Table Primers used for *hsp65* sequencing. (DOCX)

S3 Table The abundance of genera in the study population. (DOCX)

S4 Table Isolation of multiple NTM species in study subjects. (DOCX)

S1 Fig Selection of minimum sequencing depth. (PDF)

S2 Fig Structure of the NTM mock community. (PDF)

S3 Fig Mycobacterial abundance and *hsp65* amplicon concentration prior to library pooling. (PDF)

S4 Fig Genus level community structure by sample. (PDF)

S5 Fig Mycobacterial alpha diversity between controls (blue) and cases (orange). (PDF)

S6 Fig Mycobacterial alpha diversity between untreated (blue) and treated (orange) cases. (PDF)

S7 Fig Mycobacterial alpha diversity and forced expiratory volume in 1 second (FEV_1_) in NTM cases. (PDF)

S8 Fig Proportion of mycobacterial sequences and discordance between sequencing and culture. (PDF)
