# Supplementary material for: Profiling mycobacterial communities in pulmonary nontuberculous mycobacterial disease
Source: PLoS One. 2018 Dec 11;13(12):e0208018. doi: 10.1371/journal.pone.0208018 (PMC6289444; doi:10.1371/journal.pone.0208018)
Supplement: S1 File — (DOCX) [file pone.0208018.s001.docx]

**Profiling Mycobacterial Communities in Pulmonary Nontuberculous Mycobacterial Disease**

Steven A. Cowman^1,2^, Phillip James^1^, Robert Wilson^1,2^, William O. C. Cookson^1^, Miriam F. Moffatt*^1^ and Michael R. Loebinger*^1,2^.

**SUPPORTING INFORMATION**

**Supplementary methods**

**Mock community formation**

The nontuberculous mycobacteria (NTM) typestrains used in the formation of the mock community are shown in S1 Table.

Lyophilised genomic DNA was obtained from the Leibniz-Institut Deutsche Sammlung von Mikroorganismen und Zellkulturen (DSMZ, Braunschweig, Germany). For each typestrain, *hsp65* amplicons were generated by PCR using the TB11 (5’ – ACC AAC GAT GGT GTG TCC AT - 3’) and TB12 (5’ – CTT GTC GAA CCG CAT ACC CT - 3’) primers and gel-purified using the QIAquick Gel Extraction Kit (QIAGEN, Hilden, Germany). Purified amplicons were cloned into *E. coli* cells using the TOPO® TA Cloning® Kit for Sequencing (Invitrogen/Thermo Fisher Scientific, Waltham, USA) and cloned plasmid DNA extracted using the PureLink Quick Plasmid DNA Miniprep Kit (Invitrogen/Thermo Fisher Scientific). Typestrain plasmid DNA was quantified by qPCR using TB11 and TB12 primers and pooled in equimolar proportions to a final concentration 2 x 10^5^ copies/μl.

**Reference database creation**

Described NTM species were identified from the List of Prokaryotic names with Standing in Nomenclature [1] (http://www.bacterio.net - accessed 12/8/14) and *hsp65* sequences for each species were obtained from the NCBI nucleotide database (http://www.ncbi.nlm.nih.gov/nuccore/ – accessed on 13/8/2014). To identify non-mycobacterial sequences which may potentially be amplified by the TB11/TB12 primer pair, NCBI Primer-BLAST (http://www.ncbi.nlm.nih.gov/tools/primer-blast/ - accessed on 13/8/2014) was used allowing up to two mismatches at each primer site, and the *hsp65* sequences encompassed by the primer pair were retrieved. An additional 11 sequences were later added from the sequencing of *hsp65* amplicons cloned for the construction of the mock community described above giving a total of 389 sequences in the reference database. The sequences were aligned, trimmed and formatted for use with QIIME.

**Primer design**

Barcoded paired-end primers were constructed as described by Kozich et al. [2] using index sequences from the Nextera Index Kit (Illumina, San Diego, USA). Primer sequences are given in S2 Table.

**DNA extraction**

Samples were thawed within a dedicated, UV-sterilised hood and 300μl transferred into Lysing Matrix E tubes (MP Biomedicals, Solon, USA) containing 10% w/v hexadecyl-trimethyl-ammonium bromide (CTAB). Phenol:chloroform:isoamyl alcohol 25:24:1 was added and the samples were lysed and homogenised using the Precellys 24 bead beater (Bertin Technologies, Montigny le Bretonneux, France) at 6000rpm. The aqueous supernatant was then separated by the addition of chloroform:isoamyl alcohol 5:1 and centrifugation in 15ml heavy phase-lock gel tubes (5 PRIME, Hilden, Germany). DNA was precipitated by incubation overnight with linearised polyacrylamide and 30% polyethylene glycol, washed with 70% ethanol, suspended in low-EDTA TE buffer and stored at -20°C until use.

**Library preparation and sequencing**

Mycobacterial *hsp65* sequences were amplified in quadruplicate from DNA samples using the barcoded primers described above in a dedicated template-free UV irradiated hood using fresh plasticware. Each reaction contained 12.5μl Q5 High-Fidelity 2X Master Mix (New England BioLabs, Ipswich, USA), 5μl of forward primer (1.5μM), 5μl of reverse primer (1.5μM), 1.5μl water and 1μl template DNA. On each plate 1μl PCR-grade water and 1μl of mock community were then added as negative and positive controls respectively. The four plates were then amplified in parallel with the following conditions: 98°C for 30 seconds, then 35 cycles of 98°C for 10 seconds, 66°C for 30 seconds and 72°C for 5 minutes, then 73°C for 2 minutes.

Products from positive and negative control reactions from each PCR replicate were visualised on 1.5% agarose gels to confirm amplification and the four replicate plates were then pooled.

Primer-dimer sequences were removed from the pooled samples using Agencourt AMPure XP beads (Beckman Coulter, Brea, USA). Purified samples were then quantified using the Quant-iT PicoGreen dsDNA Assay Kit (Applied Biosystems/Thermo Fisher Scientific) and pooled by combining 150ng DNA from each sample. The pooled library was subject to a further round of bead-based purification using Agencourt AMPure XP beads then visualised on 1.5% agrose gel and gel-purified using the QIAquick Gel Extraction Kit (QIAGEN, Hilden, Germany). Adequate purification of the *hsp65* amplicon was confirmed by electrophoresis using the 2100 Bioanalyser and high sensitivity DNA chip (Agilent Technologies, Santa Clara, USA). The final library was quantified using the KAPA Library Quantification Kit (ROX Low) for Illumina (KAPA Biosystems, Wilmington, USA) on the Viia 7 Real-Time PCR System (Applied Biosystems/Thermo Fisher Scientific).

Sequencing was performed on the MiSeq benchtop sequencer using MiSeq v3 cartridges and reagents (Illumina) using a 12pM library and 20% spike of exogenous genomic DNA from the Phi X 174 bacteriophage (Illumina) according to the manufacturer’s instructions.

**Sequence processing**

Forward and reverse barcodes for each sequence were extracted and joined, and forward and reverse read sequences were trimmed to 250bp and adaptor sequences removed. Paired and trimmed sequences with over 200bp overlap and 10% or less mismatches were joined together. Joined sequences with a minimum mean Phred quality score of 30, at least 70% of the read consecutively above a quality score of 30 and less than 10bp of consecutive low quality scores were retained and demultiplexed. Demultiplexed sequences were aligned to the Phi-X genome and erroneous Phi-X sequences were removed.

OTUs were identified using the uclust algorithm in QIIME 1.8.0 [3,4] against the created *hsp65* reference database. Chimeric sequences with less than 60% similarity to reference sequences were removed. OTUs were clustered at ≥99% identity and closed reference based OTU picking performed. For samples that were not assigned an OTU, a 10% subsample was taken and used to form a *de novo* reference database for a new round of open reference based OTU picking, whereby samples still not identified were assigned to *de novo* OTUs. The most abundant sequence within each OTU was selected as the representative sequence. Chimeras were removed using the ChimeraSlayer algorithm [5]and taxonomies were assigned to each OTU by using the uclust algorithm. In cases where a taxonomy could not be assigned by uclust, alignment against known nucleotide sequences using the basic local alignment search tool (BLAST) (http://blast.ncbi.nlm.nih.gov/blast.cgi) was used to assign a taxonomy if possible. Finally a .BIOM format OTU table was created for analysis.

**Supplementary results**

The median Phred score was above 30 for all bases of the joined sequences.

No amplification was noted in negative controls on gel electrophoresis of *hsp65* amplification products and DNA yield was minimal in both extraction controls (3.04 and 3.32 ng/μl). A minimal number of sequences were identified in the negative control (2,656 sequences, of which only 15 were of mycobacterial origin) and the extraction controls (330 and 444 sequences, of which 18 and 41 respectively were of mycobacterial origin).

After inspection of sequencing depth per samples and rarefaction curves (Figure S1) a cut off of 5000 sequences per samples was used to identify those with a low depth of sequencing for exclusion. The proportion of the 14 excluded samples was similar between cases (24%) and controls (21%).

**Supplementary references**

1. Euzeby JP. List of Bacterial Names with Standing in Nomenclature: a folder available on the Internet. Int J Syst Bacteriol. 1997;47: 590–592.

2. Kozich JJ, Westcott SL, Baxter NT, Highlander SK, Schloss PD. Development of a dual-index sequencing strategy and curation pipeline for analyzing amplicon sequence data on the MiSeq Illumina sequencing platform. Appl Environ Microbiol. 2013;79: 5112–5120. doi:10.1128/AEM.01043-13

3. Caporaso JG, Kuczynski J, Stombaugh J, Bittinger K, Bushman FD, Costello EK, et al. QIIME allows analysis of high-throughput community sequencing data. Nat Methods. 2010;7: 335–336. doi:10.1038/nmeth.f.303

4. Edgar RC. Search and clustering orders of magnitude faster than BLAST. Bioinformatics. 2010;26: 2460–2461. doi:10.1093/bioinformatics/btq461

5. Haas BJ, Gevers D, Earl AM, Feldgarden M, Ward DV, Giannoukos G, et al. Chimeric 16S rRNA sequence formation and detection in Sanger and 454-pyrosequenced PCR amplicons. Genome Research. 2011;21: 494–504. doi:10.1101/gr.112730.110
