# Supplementary material for: Profiling mycobacterial communities in pulmonary nontuberculous mycobacterial disease
Source: PLoS One. 2018 Dec 11;13(12):e0208018. doi: 10.1371/journal.pone.0208018 (PMC6289444; doi:10.1371/journal.pone.0208018)
Supplement: S1 Table — (DOCX) [file pone.0208018.s002.docx]

| **Species** | **DSMZ code** |
| --- | --- |
| *M. psychrotolerans* | 44697 |
| *M. abscessus subsp. abscessus* | 44196 |
| *M. abscessus subsp. bolletii* | 45149 |
| *M. massiliense* | 45103 |
| *M. avium subsp. avium* | 44156 |
| *M. chelonae subsp. chelonae* | 43804 |
| *M. fortuitum subsp. fortuitum* | 46621 |
| *M. gordonae* | 44160 |
| *M. intracellulare* | 43223 |
| *M. kansasii* | 44162 |
| *M. malmoense* | 44163 |
| *M. simiae* | 44165 |
| *M. szulgai* | 44166 |
| *M. xenopi* | 43995 |
| *M. bovis* | 43990 |
| *M. peregrinum* | 43271 |
| *M. arupense* | 44942 |
| *M. rhodesiae* | 44223 |

**S1 Table. NTM typestrains included in the mock community.**
