# Supplementary material for: Profiling mycobacterial communities in pulmonary nontuberculous mycobacterial disease
Source: PLoS One. 2018 Dec 11;13(12):e0208018. doi: 10.1371/journal.pone.0208018 (PMC6289444; doi:10.1371/journal.pone.0208018)
Supplement: S2 Table — (DOCX) [file pone.0208018.s003.docx]

| **Name** | **Direction** | **Sequence** |
| --- | --- | --- |
| TB11N501 | Forward | 5'-AATGATACGGCGACCACCGAGATCTACACTAGATCGCTCATATCACGCAGACCAACGATGGTGTGTCCAT-3' |
| TB11N502 | Forward | 5'-AATGATACGGCGACCACCGAGATCTACACCTCTCTATTCATATCACGCAGACCAACGATGGTGTGTCCAT-3' |
| TB11N503 | Forward | 5'-AATGATACGGCGACCACCGAGATCTACACTATCCTCTTCATATCACGCAGACCAACGATGGTGTGTCCAT-3' |
| TB11N504 | Forward | 5'-AATGATACGGCGACCACCGAGATCTACACAGAGTAGATCATATCACGCAGACCAACGATGGTGTGTCCAT-3' |
| TB11N505 | Forward | 5'-AATGATACGGCGACCACCGAGATCTACACGTAAGGAGTCATATCACGCAGACCAACGATGGTGTGTCCAT-3' |
| TB11N506 | Forward | 5'-AATGATACGGCGACCACCGAGATCTACACACTGCATATCATATCACGCAGACCAACGATGGTGTGTCCAT-3' |
| TB11N507 | Forward | 5'-AATGATACGGCGACCACCGAGATCTACACAAGGAGTATCATATCACGCAGACCAACGATGGTGTGTCCAT-3' |
| TB11N508 | Forward | 5'-AATGATACGGCGACCACCGAGATCTACACCTAAGCCTTCATATCACGCAGACCAACGATGGTGTGTCCAT-3' |
| TB12N701 | Reverse | 5'-CAAGCAGAAGACGGCATACGAGATTAAGGCGAATGAGATCATTGGCTTGTCGAACCGCATACCCT-3' |
| TB12N702 | Reverse | 5'-CAAGCAGAAGACGGCATACGAGATCGTACTAGATGAGATCATTGGCTTGTCGAACCGCATACCCT-3' |
| TB12N703 | Reverse | 5'-CAAGCAGAAGACGGCATACGAGATAGGCAGAAATGAGATCATTGGCTTGTCGAACCGCATACCCT-3' |
| TB12N704 | Reverse | 5'-CAAGCAGAAGACGGCATACGAGATTCCTGAGCATGAGATCATTGGCTTGTCGAACCGCATACCCT-3' |
| TB12N705 | Reverse | 5'-CAAGCAGAAGACGGCATACGAGATGGACTCCTATGAGATCATTGGCTTGTCGAACCGCATACCCT-3' |
| TB12N706 | Reverse | 5'-CAAGCAGAAGACGGCATACGAGATTAGGCATGATGAGATCATTGGCTTGTCGAACCGCATACCCT-3' |
| TB12N707 | Reverse | 5'-CAAGCAGAAGACGGCATACGAGATCTCTCTACATGAGATCATTGGCTTGTCGAACCGCATACCCT-3' |
| TB12N708 | Reverse | 5'-CAAGCAGAAGACGGCATACGAGATCAGAGAGGATGAGATCATTGGCTTGTCGAACCGCATACCCT-3' |
| TB12N709 | Reverse | 5'-CAAGCAGAAGACGGCATACGAGATGCTACGCTATGAGATCATTGGCTTGTCGAACCGCATACCCT-3' |
| TB12N710 | Reverse | 5'-CAAGCAGAAGACGGCATACGAGATCGAGGCTGATGAGATCATTGGCTTGTCGAACCGCATACCCT-3' |
| TB12N711 | Reverse | 5'-CAAGCAGAAGACGGCATACGAGATAAGAGGCAATGAGATCATTGGCTTGTCGAACCGCATACCCT-3' |
| TB12N712 | Reverse | 5'-CAAGCAGAAGACGGCATACGAGATGTAGAGGAATGAGATCATTGGCTTGTCGAACCGCATACCCT-3' |
| Read 1 Primer | | 5’‑TCATATCACGCAGACCAACGATGGTGTGTCCAT‑3’ |
| i7 Index Primer | | 5’‑AGGGTATGCGGTTCGACAAGCCGATGATCTAAT‑3’ |
| Read 2 Primer | | 5’‑ATGAGATCATTGGCTTGTCGAACCGCATACCCT‑3’ |

**S2 Table. Primers used for *hsp65* sequencing.**

Oligonucleotide sequences © 2007-2013 Illumina, Inc. All rights reserved. Derivative works created by Illumina customers are authorized for use with Illumina instruments and products only. All other uses are strictly prohibited
