# Supplementary material for: Profiling mycobacterial communities in pulmonary nontuberculous mycobacterial disease
Source: PLoS One. 2018 Dec 11;13(12):e0208018. doi: 10.1371/journal.pone.0208018 (PMC6289444; doi:10.1371/journal.pone.0208018)
Supplement: S3 Table — (DOCX) [file pone.0208018.s004.docx]

| **Genus** | **Raw sequences** | **Proportion of total sequences** | **Mean relative abundance per sample** | **No. subjects where dominant** | **Number of OTUs** |
| --- | --- | --- | --- | --- | --- |
| *Actinomyces* | 2118108 | 51.10% | 50.36% | 24 | 1515 |
| *Rothia* | 1440743 | 34.76% | 34.23% | 14 | 1526 |
| *Mycobacterium* | 258028 | 6.23% | 4.69% | 2 | 355 |
| *Unidentified* | 138637 | 3.34% | 1.27% | 0 | 437 |
| *Nocardia* | 102940 | 2.48% | 3.46% | 1 | 75 |
| *Bifidobacterium* | 84611 | 2.04% | 3.13% | 1 | 42 |
| *Streptomyces* | 1083 | 0.03% | 0.30% | 0 | 1 |
| *Propionibacterium* | 847 | 0.02% | <0.01% | 0 | 4 |
| *Nocardioides* | 14 | <0.01% | <0.01% | 0 | 4 |
| *Rhodococcus* | 6 | <0.01% | <0.01% | 0 | 1 |
| *Gordonia* | 2 | <0.01% | <0.01% | 0 | 1 |

**S3 Table:** **The abundance of genera in the study population.**
