# Supplementary material for: Profiling mycobacterial communities in pulmonary nontuberculous mycobacterial disease
Source: PLoS One. 2018 Dec 11;13(12):e0208018. doi: 10.1371/journal.pone.0208018 (PMC6289444; doi:10.1371/journal.pone.0208018)
Supplement: S4 Table — (DOCX) [file pone.0208018.s005.docx]

| **Diagnosed species** | **Diagnosed species detected by sequencing** | **Other isolated species** | **Other isolated species detected by sequencing** |
| --- | --- | --- | --- |
| *M. abscessus* complex | Yes | MAC | Yes |
| MAC | No | *M. xenopi* | Yes |
| *M. abscessus* complex | Yes | MAC | Yes |
| *M. simiae** | Yes | MAC*, *M. fortuitum*, unidentified | Yes (MAC, *M. fortuitum*) |
| *M. kansasii* | Yes | Unidentified | - |
| *M. kansasii* | Yes | MAC, *M. celatum* | Yes (MAC) |
| *M. malmoense* | No | *M. kansasii, M. xenopi*, MAC | No |
| MAC | Yes | *M. xenopi* | No |
| *M. kansasii* | Yes | *M. gordonae, M. chelonae, M. fortuitum* | No |
| MAC | Yes | *M. xenopi* | Yes |
| MAC | Yes | *M. abscessus* complex, *M. fortuitum* | Yes (*M. fortuitum*) |
| MAC | Yes | *M. abscessus* complex | No |
| MAC | No | *M. xenopi, M. fortuitum*, unidentified | No |

**S4 Table.** **Isolation of multiple NTM species in study subjects.** Diagnosed species refers to the species isolated in culture leading to the original diagnosis of pulmonary NTM disease. Other isolated species refers to any other NTM species isolated at any time. * isolated MAC prior to *M. simiae* but not re-isolated for 10 years
