# Supplementary material for: Profiling mycobacterial communities in pulmonary nontuberculous mycobacterial disease
Source: PLoS One. 2018 Dec 11;13(12):e0208018. doi: 10.1371/journal.pone.0208018 (PMC6289444; doi:10.1371/journal.pone.0208018)
Supplement: S1 Fig — (PDF) [file pone.0208018.s006.pdf]

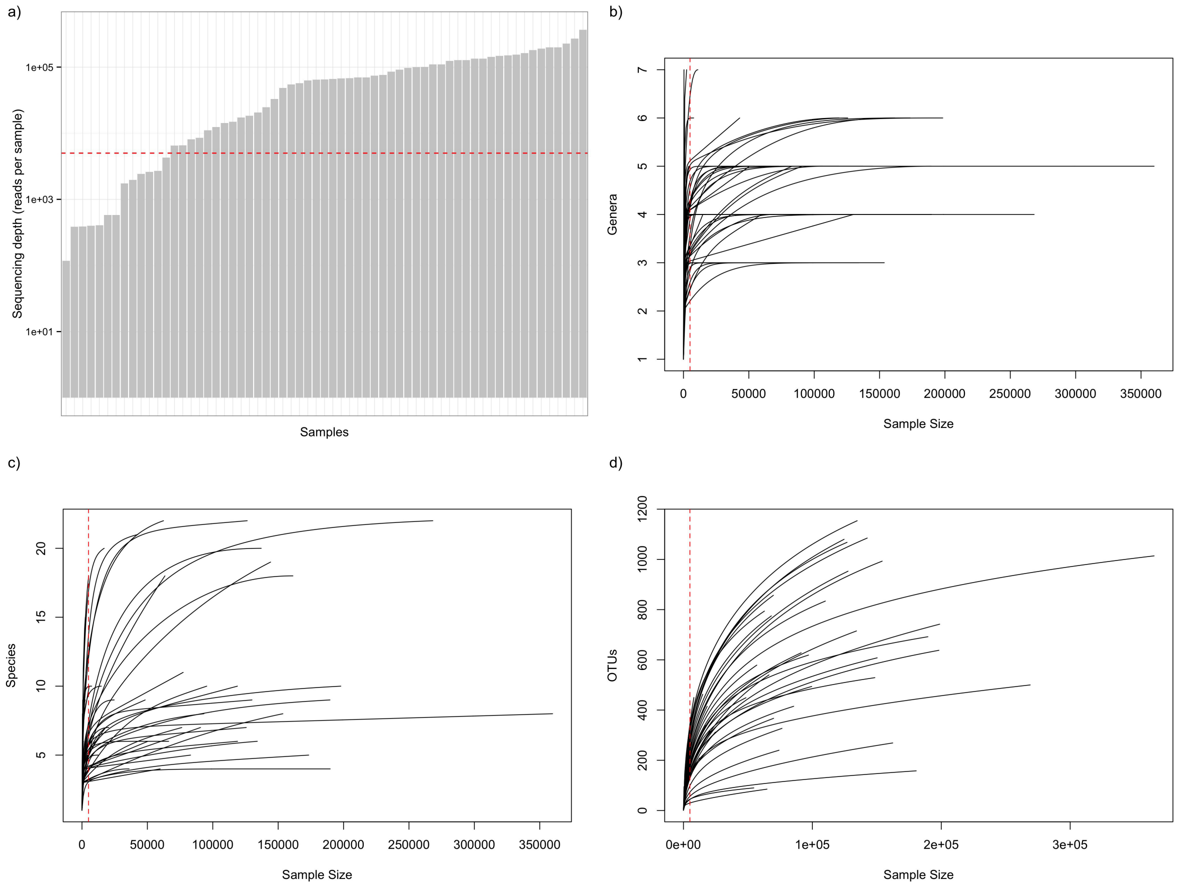

S1 Fig. Selection of minimum sequencing depth. A) Sequencing depth per sample; X-axis = samples, Y-axis = number of sequences per samples, dotted line = 5000 sequences. B,C,D) Rarefaction curves at Genus, Species and OUT level respectively; X-axis = Sequencing depth, Y-axis = Number of taxa, dotted line = 5000 sequences
