# Supplementary material for: Profiling mycobacterial communities in pulmonary nontuberculous mycobacterial disease
Source: PLoS One. 2018 Dec 11;13(12):e0208018. doi: 10.1371/journal.pone.0208018 (PMC6289444; doi:10.1371/journal.pone.0208018)
Supplement: S2 Fig — (PDF) [file pone.0208018.s007.pdf]

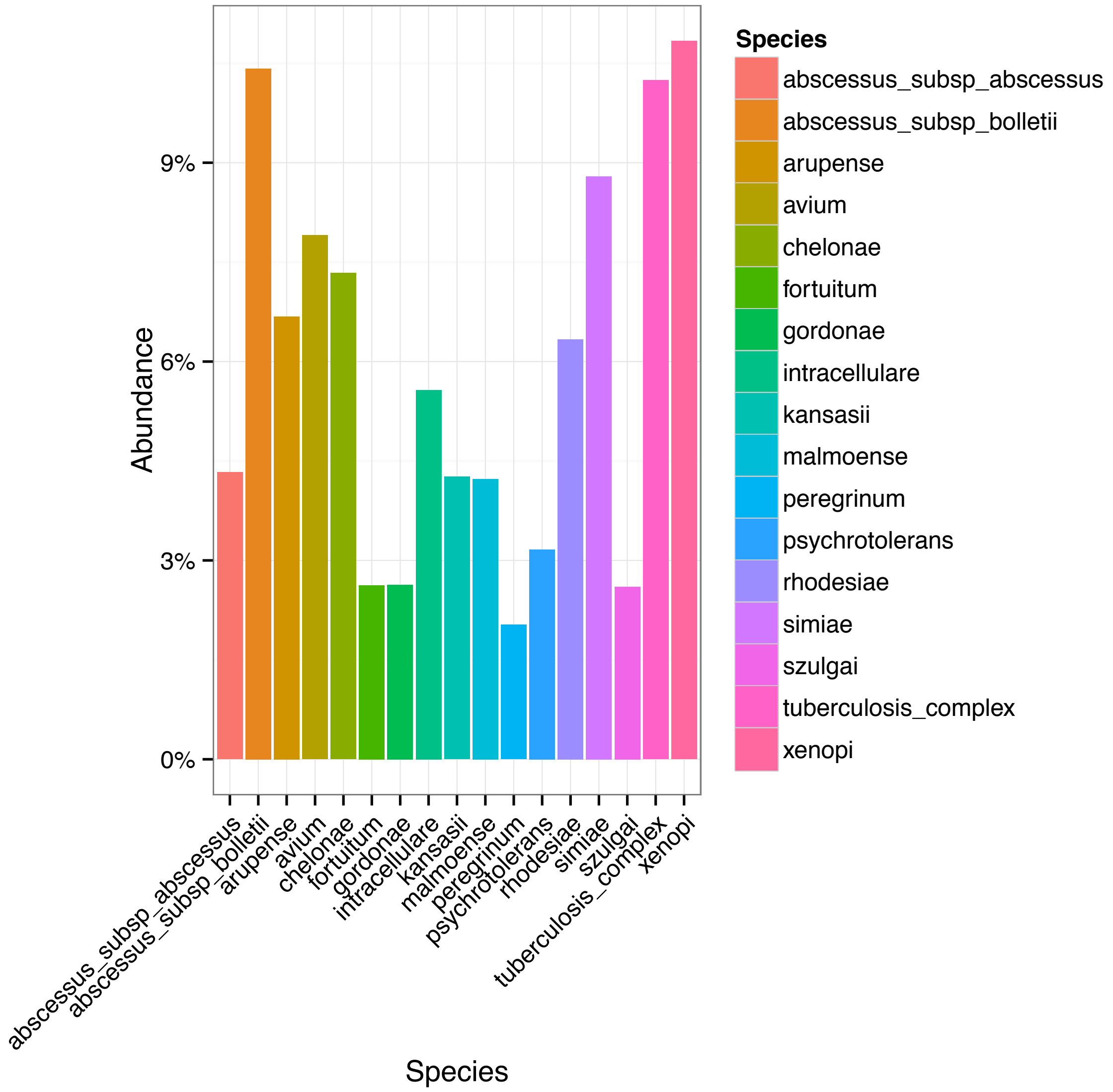


**S2 Fig: Structure of the NTM mock community.** X-axis = Mycobacterial species, Y-axis = relative abundance of each species. The expected relative abundance of each species was 11.1% for *M. abscessus subsp. bolletii* and 5.6% for all others.
