# Supplementary material for: Profiling mycobacterial communities in pulmonary nontuberculous mycobacterial disease
Source: PLoS One. 2018 Dec 11;13(12):e0208018. doi: 10.1371/journal.pone.0208018 (PMC6289444; doi:10.1371/journal.pone.0208018)
Supplement: S3 Fig — (PDF) [file pone.0208018.s008.pdf]

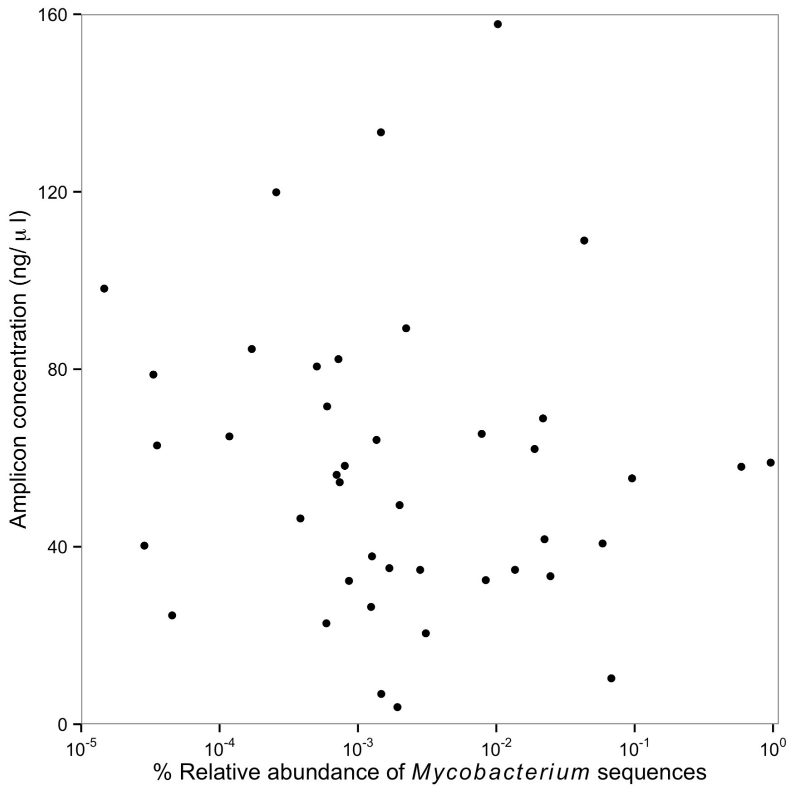

S3 Fig. Mycobacterial abundance and *hsp65* amplicon concentration prior to library pooling. X-axis = relative abundance of Mycobacterium sequences (%); Y-axis = hsp65 amplicon concentration (ng/µl).
