# Supplementary material for: Profiling mycobacterial communities in pulmonary nontuberculous mycobacterial disease
Source: PLoS One. 2018 Dec 11;13(12):e0208018. doi: 10.1371/journal.pone.0208018 (PMC6289444; doi:10.1371/journal.pone.0208018)
Supplement: S4 Fig — (PDF) [file pone.0208018.s009.pdf]

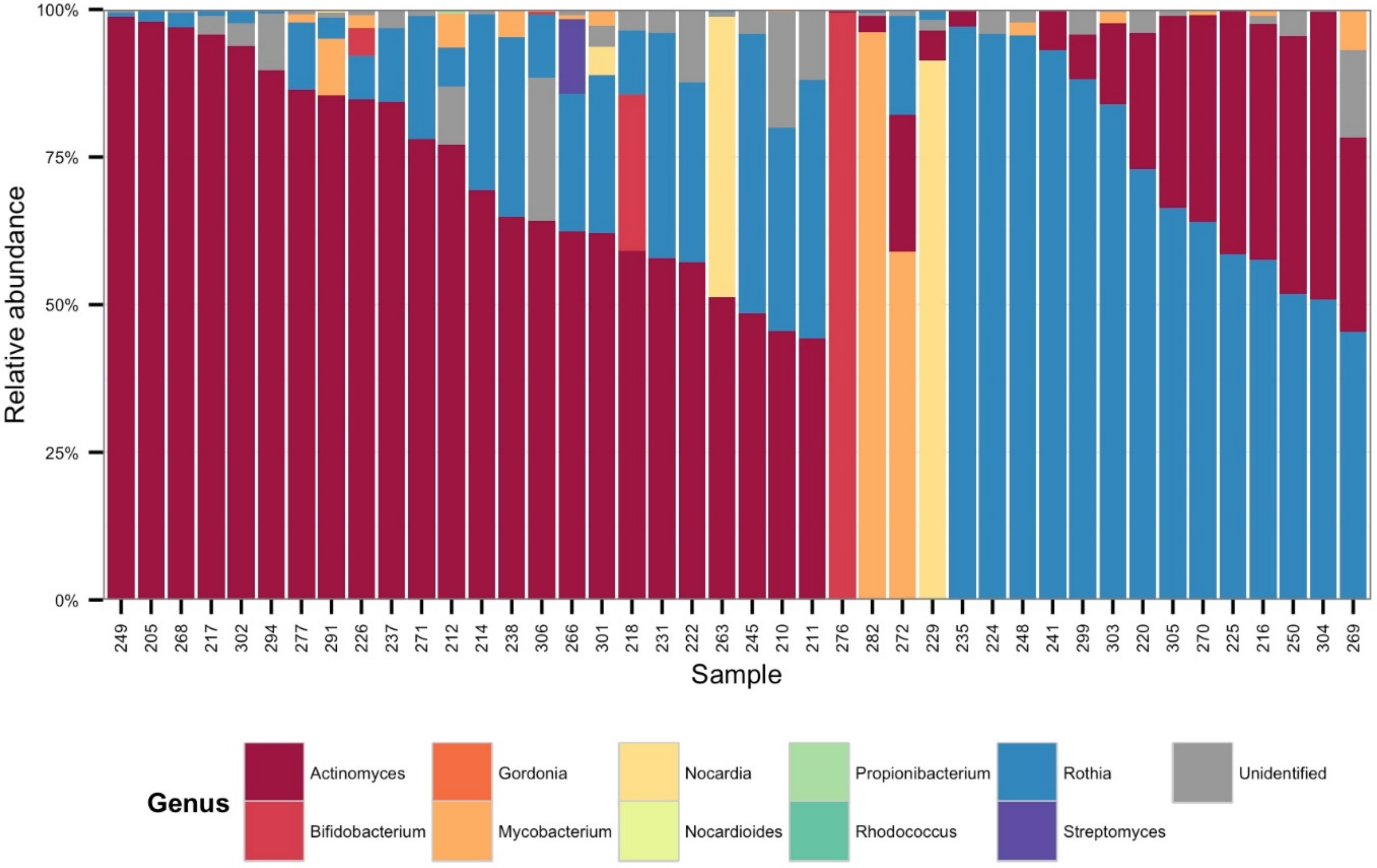

S4 Fig: Genus level community structure by sample. X-axis = samples, y-axis = proportional abundance, colours = genera as indicated by key.
