# Supplementary material for: Profiling mycobacterial communities in pulmonary nontuberculous mycobacterial disease
Source: PLoS One. 2018 Dec 11;13(12):e0208018. doi: 10.1371/journal.pone.0208018 (PMC6289444; doi:10.1371/journal.pone.0208018)
Supplement: S5 Fig — (PDF) [file pone.0208018.s010.pdf]

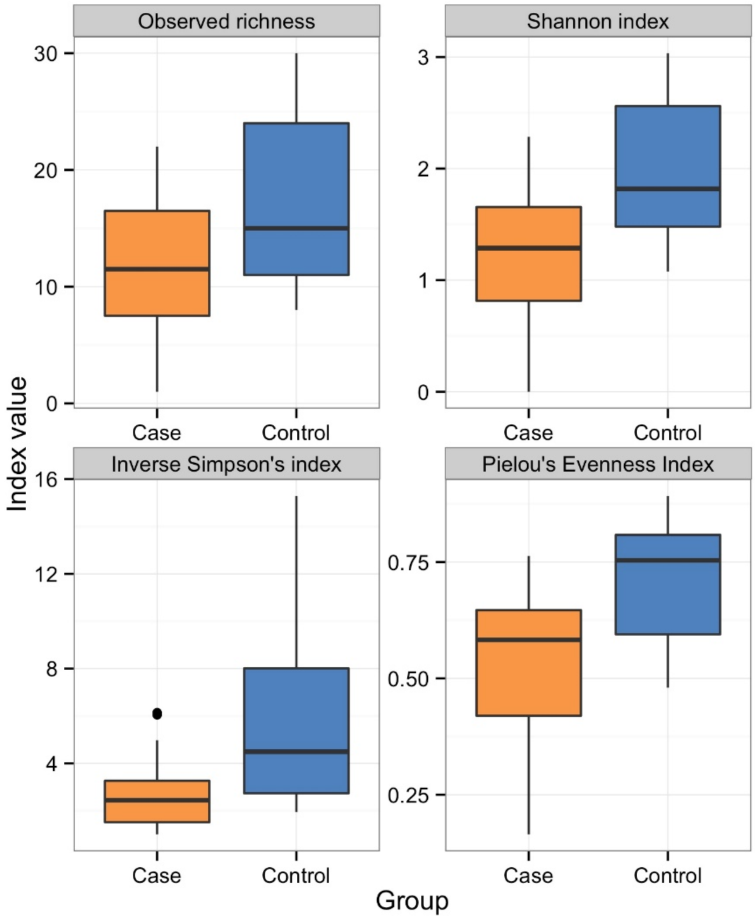

S5 Fig: Mycobacterial alpha diversity between controls (blue) and cases (orange). X-axis = study group, Y-axis = diversity index value. Shannon Index (*P* = 0.05) and Pielou's Evenness Index (*P* = 0.028) were significantly lower in cases; there was no significant difference in Observed richness (*P* = 0.166) or Inverse Simpson's Index (*P* = 0.109).
