# Supplementary material for: Profiling mycobacterial communities in pulmonary nontuberculous mycobacterial disease
Source: PLoS One. 2018 Dec 11;13(12):e0208018. doi: 10.1371/journal.pone.0208018 (PMC6289444; doi:10.1371/journal.pone.0208018)
Supplement: S6 Fig — (PDF) [file pone.0208018.s011.pdf]

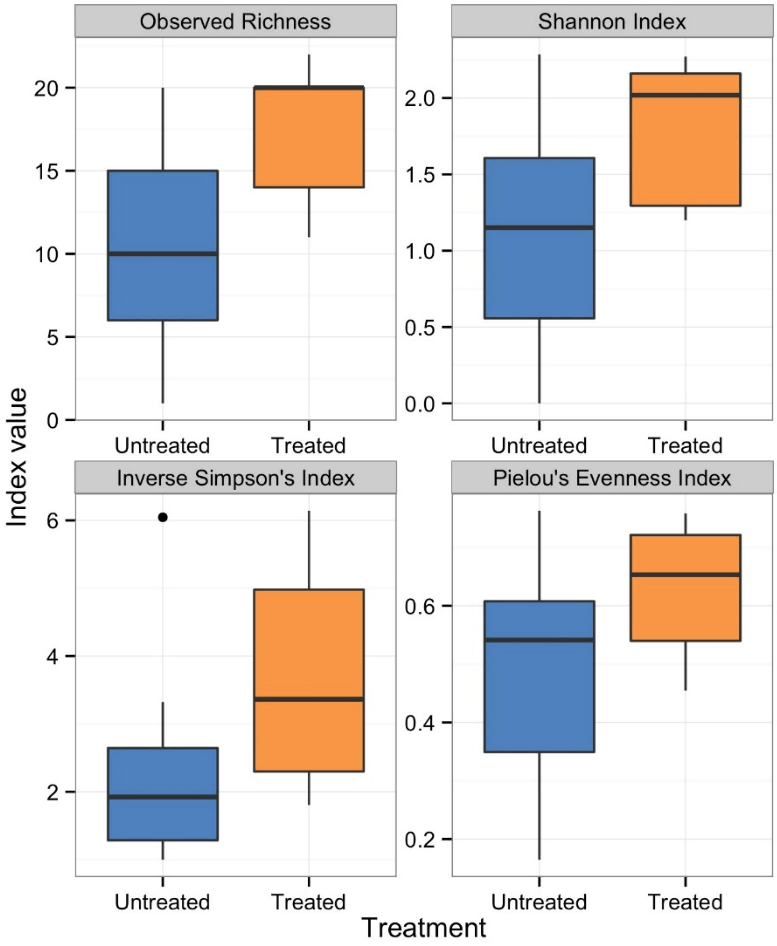


**S6 Fig: Mycobacterial alpha diversity between untreated (blue) and treated (orange) cases.** X-axis = treatment group, Y-axis = diversity index value. Shannon Index ($P$ = 0.035) and Observed richness ($P$ = 0.021) were significantly lower in cases; there was no difference in Pielou's Evenness Index ($P$ = 0.091) or Inverse Simpson's Index ($P$ = 0.166).
