# Supplementary material for: Profiling mycobacterial communities in pulmonary nontuberculous mycobacterial disease
Source: PLoS One. 2018 Dec 11;13(12):e0208018. doi: 10.1371/journal.pone.0208018 (PMC6289444; doi:10.1371/journal.pone.0208018)
Supplement: S7 Fig — (PDF) [file pone.0208018.s012.pdf]

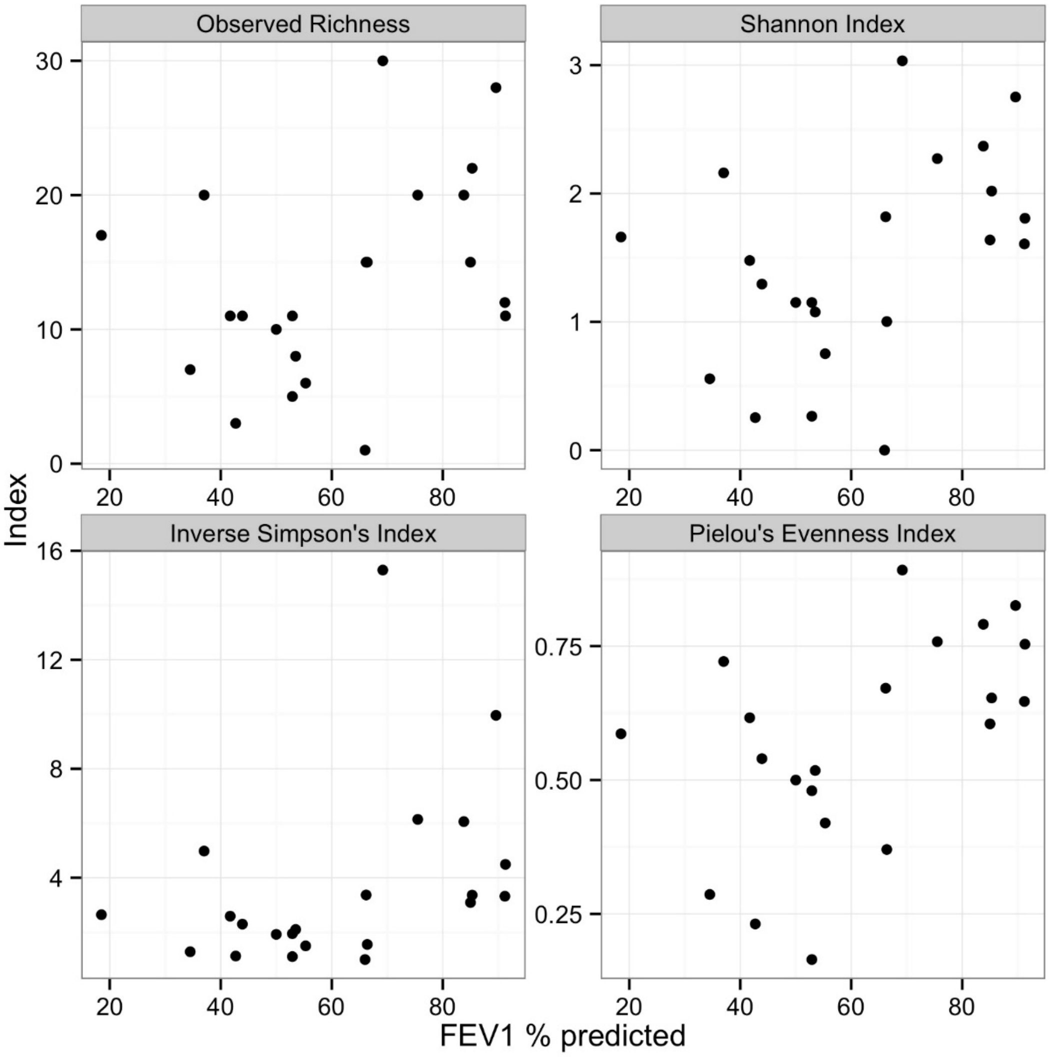

S7 Fig: Mycobacterial alpha diversity and forced expiratory volume in 1 second (FEV_1) in NTM cases. X-axis = FEV_1 % predicted, Y-axis = diversity index value. There was a significant positive correlation for Shannon Index (r = 0.43 (95% CI 0.01 to 0.72), P = 0.044) and Pielou's Evenness Index (r = 0.51 (95% CI 0.10 to 0.77), P = 0.018) but not for Observed richness (r = 0.37 (95% CI -0.06 to 0.69), P = 0.09) or Inverse Simpson's Index (r = 0.07 (95% CI -0.04 to 0.70), P = 0.39).
