# Supplementary material for: Profiling mycobacterial communities in pulmonary nontuberculous mycobacterial disease
Source: PLoS One. 2018 Dec 11;13(12):e0208018. doi: 10.1371/journal.pone.0208018 (PMC6289444; doi:10.1371/journal.pone.0208018)
Supplement: S8 Fig — (PDF) [file pone.0208018.s013.pdf]

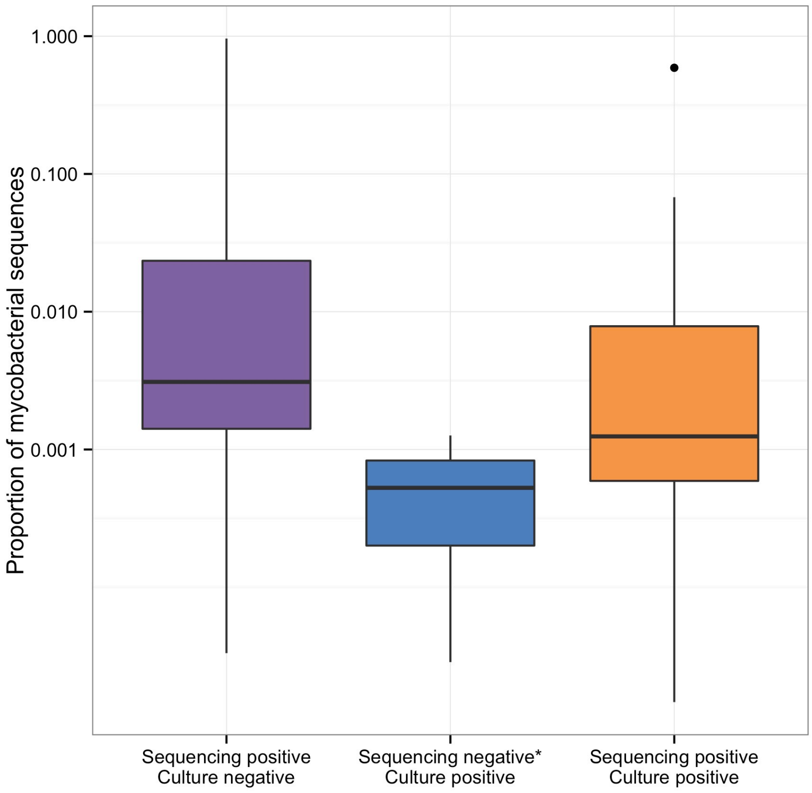

S8 Fig: Proportion of mycobacterial sequences and discordance between sequencing and culture. X-axis = sequencing/culture discordance, Y-axis = Proportion of mycobacterial sequence. * refers to samples where the cultured NTM was not detected by sequencing.
